# Measurement of optical density of microbes by multi‐light path transmission method

**DOI:** 10.1002/mlf2.12147

**Published:** 2024-12-01

**Authors:** Hongwei Wang, Carina M. Gu, Sujuan Xu, Hongfeng Wang, Xiaomin Zhao, Lichuan Gu

**Affiliations:** ^1^ State Key Laboratory of Microbial Technology Shandong University Qingdao China; ^2^ The Faculty of Science University of British Columbia Vancouver British Columbia Canada; ^3^ Shandong Peanut Research Institute Qingdao China

**Keywords:** Beer–Lambert law, cell density, growth curve, microbe, optical density

## Abstract

Optical density (OD) is an important indicator of microbial density, and a commonly used variable in growth curves to express the growth of microbial culture. However, OD values show a linear relationship with bacterial concentration only at low concentrations. When the cell density is high, the relationship loses linearity, and serial dilution is needed to obtain readings of better accuracy. Here, we show that measuring OD values using shorter light paths is in close equivalence to measuring OD values of the cell culture with corresponding dilution. By measuring three different light paths simultaneously, accurate OD values can be easily obtained from low to high cell density. Using this method, growth curves of *Escherichia coli*, *Staphylococcus aureus*, and *Pichia pastoris* are measured with higher accuracy. To further simplify the process, an l‐shaped cuvette and a corresponding turbidimeter are designed specifically for OD value measurement based on the multi‐light path transmission method.

## Impact statement

Although the measurement of microbial cell density is one of the simplest experiments, it has troubled microbiologists and related industries for more than half a century. An ideal measurement technique should have the features of high accuracy, high throughput, easy operation, and noninvasion. The current method cannot even meet the requirements of high accuracy and high throughput at the same time. Multi‐light path transmission method, however, is a solid step toward this goal. The solution to this problem will certainly help improve the efficiency of microbial experiments and promote the development of the fermentation industry by reducing costs and improving production and quality.

## INTRODUCTION

Measurements of microbial density have widespread usage in both microbiology research and related bio‐industry. Optical density (OD) is often used to express the turbidity of microbial cultures, which is a representation of the cell density. Standard OD readings are obtained from spectrophotometers by detecting a light source of around 600 nm wavelength (OD_600_) after passing through 10 mm of suspension^1^, ^2^. The principle behind this method is the Beer–Lambert law, which states the relationship between the molar concentration of the solute in the uniformly distributed solutions and the absorbance of light as *A* = *ϵcl*, where *c* is the molar concentration, *ϵ* is the molar extinction coefficient, and *A* is the absorbance^3^. The term optical density is used in place of absorbance when describing microbial densities as uniformly distributed cell particles in suspensions scatter in addition to absorbing light. Spectrophotometers are ideal for measuring microbial concentrations as they provide simple, efficient, and inexpensive OD readings.

However, the ratio of OD readings to cell densities is approximately constant only at relatively low cell densities. As the cells multiply and their density becomes higher, the relationship between OD readings and the cell densities gradually deviates from linearity. Since light deflected away from the beam by one particle can be scattered back by another particle, OD readings (apparent OD) could be much lower than they should be at high turbidities. This makes it possible for cell densities to be significantly underestimated^4^, ^5^.

Many efforts have been made to deal with the problem that Beer–Lambert law does not apply to high cell‐density conditions. A recent study reported that a plate reader calibrated using silica microspheres showed higher precision^6^. Laser speckle imaging was used to perform real‐time monitoring of bacteria growth kinetic in liquid culture media^7^. Deep‐learning AI‐based live tracking of bacterial growth also exhibited the potential for improved measurement^8^. The most common practice in microbiology labs is to dilute the cell culture before measurement when accurate OD values are required at high turbidities.

In this study, we found that the OD values measured with the shorter light paths were equivalent to those mesured with the corresponding dilution method. Based on this discovery, ODs of three different light paths were measured and combined as a weighted average, accurately measuring OD values in a large range from low to high density. Using this method, growth curves of *Escherichia coli*, *Staphylococcus aureus*, and *Pichia pastoris* were measured with higher accuracy than using only a 10 mm light path. To achieve enhanced process simplification, an L‐shaped cuvette and its corresponding turbidimeter were designed for OD value measurement via the multi‐light path transmission approach.

## RESULTS

### Shorter light paths are equivalent to corresponding dilutions in the measurement of OD_600_

**Figure 1:**
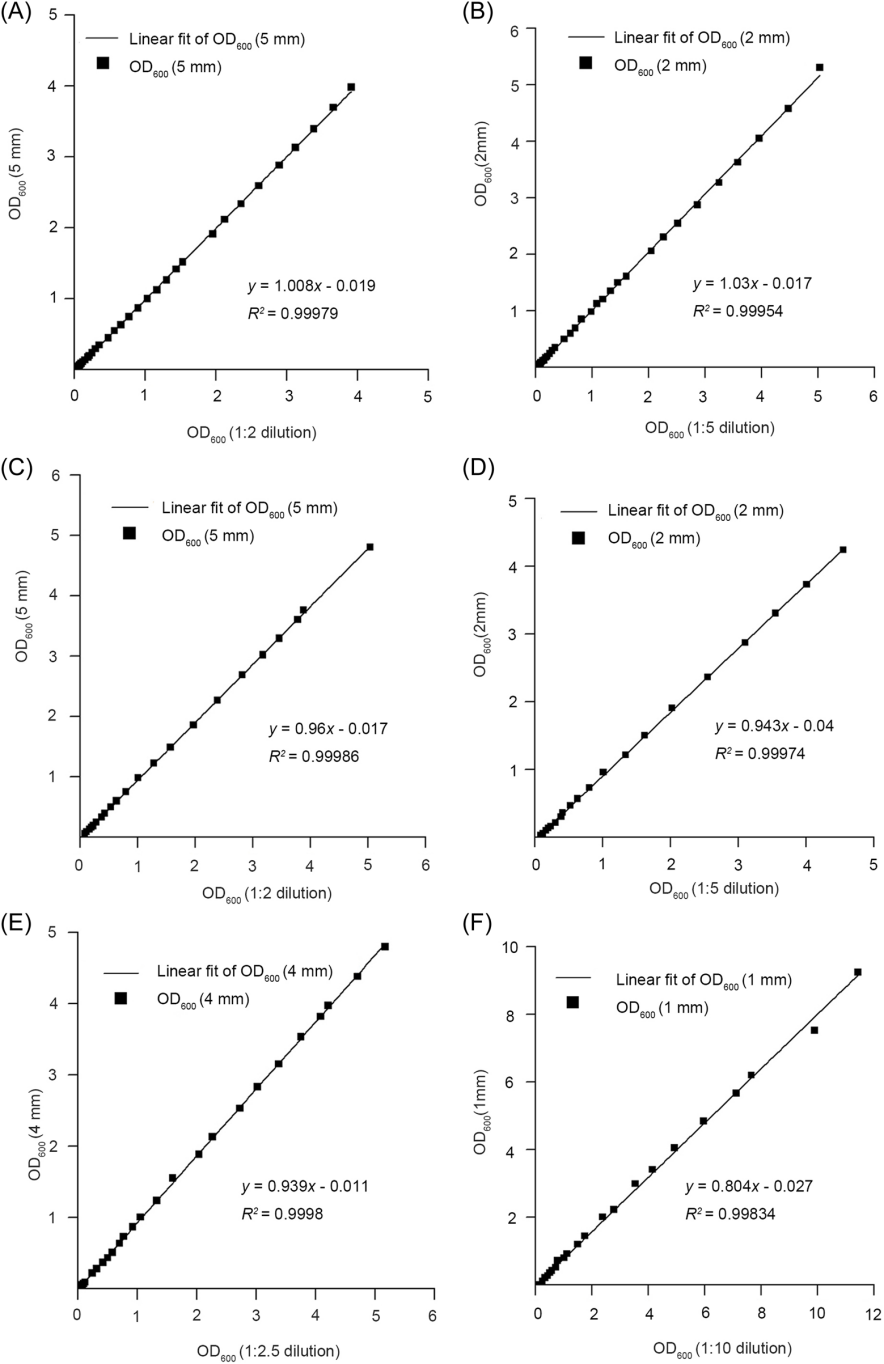
The shorter light path method is equivalent to the dilution method. (A, B) The OD_600_ values of *Escherichia coli* culture obtained from 1:2 and 1:5 dilution are linearly correlated with 5 and 2 mm light paths, respectively, with a slope ≈1.0. (C, D) The OD_600_ values of *Staphylococcus aureus* culture obtained from 1:2 and 1:5 dilution are also linearly correlated with 5 and 2 mm light paths, respectively, with approximately a 1.0 slope. (E, F) The OD_600_ values of *Pichia pastoris* obtained from 1:2.5 and 1:10 dilutions are linearly correlated with 4 and 1 mm light paths, respectively, with a slope <1.0. Each experiment was performed in triplicate, yielding consistent linear equations (The second and third repeats are shown as Figures S1 and S2).

Overnight culture of *E. coli* with OD_600_ of 2.431 was first serially diluted to produce a series of samples to be tested. When measured on a standard spectrophotometer, these samples gave an OD_600_ ranging from 0.007 to 2.431. Each sample was then further split into two groups and measured in different ways: the samples of the first group were further diluted two times and five times, respectively, and measured on the spectrophotometer in a cuvette with a 10 mm light path. The samples of the other group were measured on the spectrophotometer in cuvettes with 5 and 2 mm light paths, respectively. The OD_600_ values obtained by different methods clearly indicated that low cell‐density measurements with a 10 mm light path cuvette and without dilution gave a better accuracy than both dilutions and shorter light path methods. Data obtained by the latter two methods showed some fluctuation and even gave zero value at the lowest density. This is understandable as the signal at low density is weak and both dilution and shorter light path result in an even weaker signal that may approach or be below the detection limit of the spectrophotometer. At higher densities, however, the latter two methods began to give better results since the standard method obviously underestimated the OD_600_. The equivalence between dilutions and their corresponding shorter light paths is striking. Double dilutions always gave a result very close to that of the 5 mm light paths, which are just one‐half of the 10 mm light path. Quintuple dilutions also gave a result very close to that of the 2 mm light paths, which are just one‐fifth of the 10 mm light path. Equivalence between the two measurement methods was also observed when we studied *S. aureus*, a Gram‐positive bacterium. These data strongly suggest that the shorter light path method can readily replace the dilution method to measure the OD_600_ of the high‐density bacterial culture to save time and labor (Figures 1A–D, S1A–D, S2A–D, and Tables S1 and S2).

Previous studies have shown that cell size is also an important factor affecting the measurement of OD_600_. To make clear if the equivalence of the dilutions and the corresponding shorter light paths also apply to the cells of larger size, we measured OD_600_ of *P. pastoris* cultures just as we did with *E. coli*. Since the preliminary experiment indicated that the deviation from linearity at high densities was much more serious for *P. pastoris* than for *E. coli*, higher dilution is necessary. We decided to use 2.5× and 10× dilution and the corresponding 4 and 1 mm light paths. In these cases, the equivalence of the dilutions and the corresponding shorter light paths are roughly held. The shorter light path readings were slightly smaller than those of the corresponding dilutions. This, however, could be easily corrected by multiplying by a coefficient since the relationship is linear. With this correction, the shorter light path method can also replace the dilution method to measure the OD_600_ of the high‐density culture of larger cell size (Figures 1E,F, S1E,F, S2E,F, and Table S3).

### The weighted average OD of the three light paths represents the accurate OD_600_ from low to high cell densities

The results above clearly indicate that it would be ideal to use the method of a shortened light path when measuring higher densities of cell cultures. But there remains the question of when we should change the length of the light path. If the change begins at a specific point on a growth curve, there will be a jump or gap between the data obtained before and after the light path is changed. For the graph to be smoothened, we decided that the best strategy is to measure the ODs of three different light paths and calculate their weighted average. The OD value of the longest path (usually 10 mm) will be of the highest importance at lower densities and decreases as density increases. Consecutively, data measured from the path of middle length will obtain the highest importance when the density is in the midrange and the ODs of the shortest light path at higher densities. To fulfill this purpose, we formulate equations as follows:

$$\begin{array}{l} w_{1}=X_{1}/\left(X_{1}+b{X_{2}}^{E}+c{X_{3}}^{F}\right)\\ w_{2}=b{X_{2}}^{E}/\left(X_{1}+b{X_{2}}^{E}+c{X_{3}}^{F}\right)\\ w_{3}=c{X_{3}}^{F}/\left(X_{1}+b{X_{2}}^{E}+c{X_{3}}^{F}\right)\\ \mathrm{WOD}=w_{1}X_{1}+w_{2}X_{2}+w_{3}X_{3}, \end{array}$$

*w*_1_, *w*_2_, and *w*_3_ represent the weight of each light path, respectively. *X*_1_, *X*_2_, and *X*_3_ represent the OD values obtained with each light path, and *b*, *c*, *E*, and *F* are empirical coefficients based on the data obtained for a specific microbe. The weighted average OD (WOD) value is then calculated by using the last equation. For *E. coli* culture, if we have *b* = 4, *c* = 8, *E* = 2, and *F* = 3, the weighted averages are easily calculated. The results clearly showed that at low densities the WOD values were very close to the 10 mm light path readings and that at high densities they approached closely to the value of the 2 mm light path readings. In the case of *P. pastoris* cultures, the deviation from linearity is more serious at high densities, thus we need a faster‐growing weight for the 1 mm light path. We set *b* = *c* = 4, *E* = 2, *F* = 4 and calculated the weighted averages from low through high densities. Again, the results unambiguously suggest that our strategy also applies to the culture of larger cell size.

### The WOD value of the three light paths is linearly correlated with dry weight

**Figure 2:**
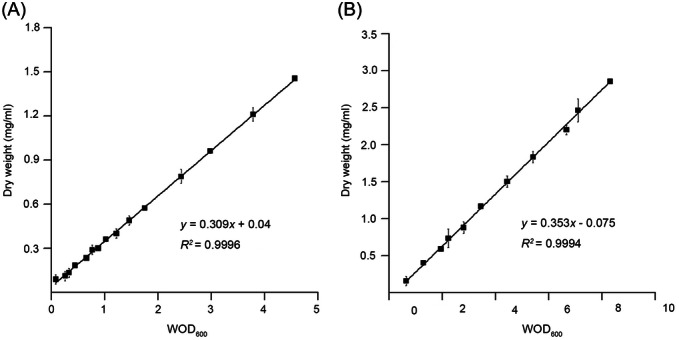
The linear relationship between WOD_600_ value and dry weight. The WOD_600_ values of *Escherichia coli* (A) and *Pichia pastoris* (B) of the three light paths (or WOD_600_) are linearly correlated with dry weight. Each experiment was performed in triplicate; means and standard deviations were calculated. WOD_600_, weighted average OD_600_.

It was reported that the dry weight of the bacteria can reflect the concentration of the bacteria^9^. To verify that the WOD_600,_ which means OD_600_ value obtained from the weighted average of the three light paths of 10 mm, 5 mm, and 2 mm, can accurately reflect the concentration of bacteria, the dry weight and the corresponding WOD_600_ were measured. Plotting these WOD values against the dry weight gave a straight line, which indicated that the WOD_600_ value can accurately detect the concentration of bacteria (Figure 2).

### The WOD of the three light paths gives much better growth curves for both *E*. *coli* and *P. pastoris*

**Figure 3:**
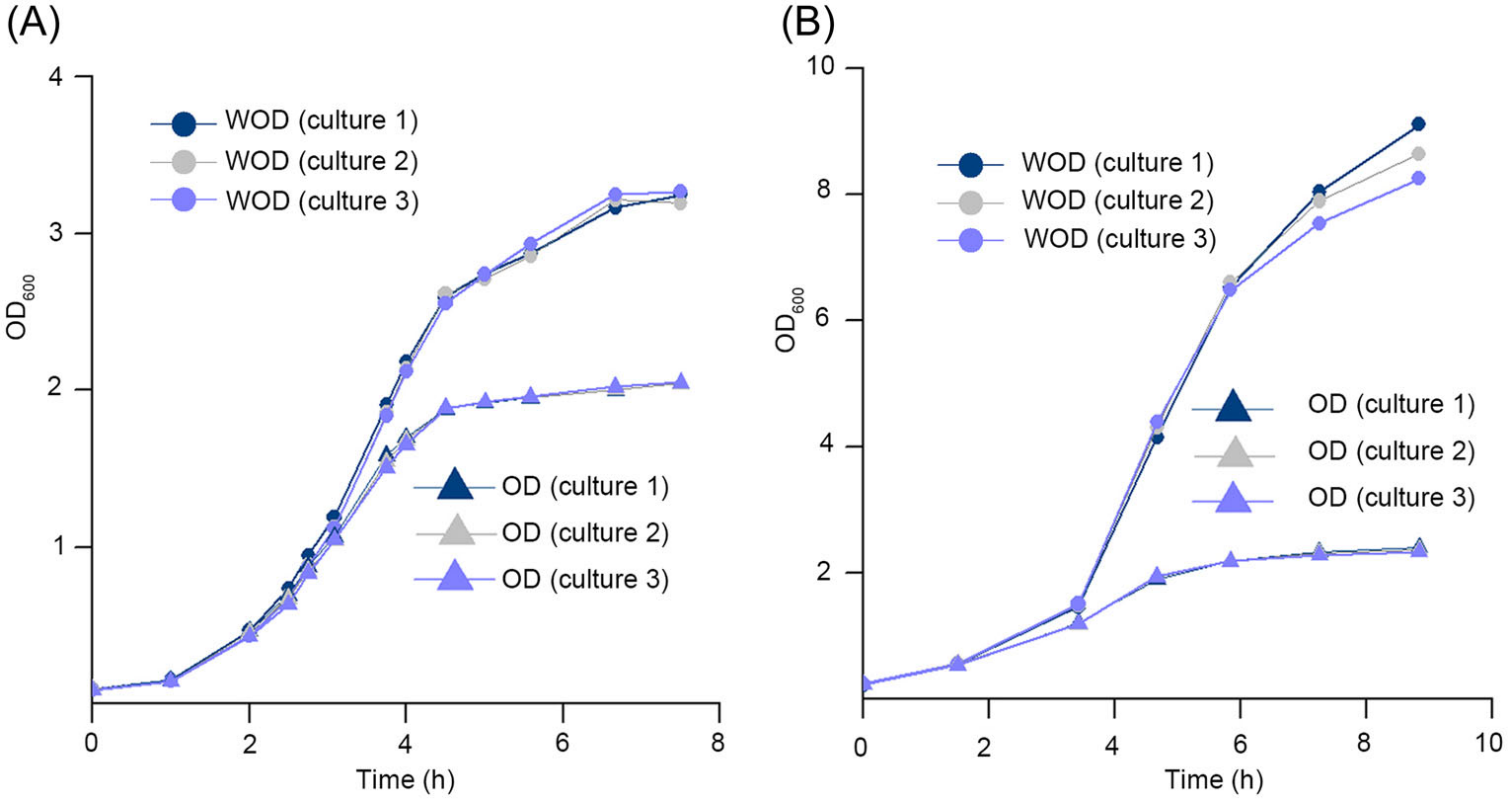
The WOD value of the three light paths shows a more reliable growth curve. (A, B) Growth curves based on the WOD value of the three light paths and OD value of 10mm light path of *E. coli* (A) and *P. pastoris* (B). The OD group shows the OD values of the three cultures (cultures 1–3) of *E. coli* (A) and *P. pastoris* (B) detected directly by using the curette with a light path of 10 mm. The WOD group shows the WOD values of the cultures obtained by using the cuvettes with the light path of 10, 5, and 2 mm.

Monitoring of microbial growth curves is one of the most common practices in microbiology laboratories. To determine if the multi‐light path transmission method gives a more accurate result of the growth curve, the WOD_600_ of three growing *E. coli* cultures was measured using 10, 5, and 2 mm cuvettes through an 8‐h time span. Growth curves were then plotted based on the WOD value of the three light paths and the OD value of 10 mm path alone, respectively (Figure 3A). As expected, growth curves obtained by the two methods overlap well at low cell densities but gradually separate when the cell density gets higher. Graphs measured using the 10 mm cuvette exhibit an earlier and much lower stationary phase. Graphs obtained from the WOD value of the three light paths suggest that the actual stationary stage begins later at a much higher OD_600_. Two groups of curves begin to deviate at an OD_600_ of 1.0, which means that for an OD_600_ higher than 1.0 to be measured accurately, the suspension must be diluted or measured using a shorter light path. The 10 mm cuvette only measured OD_600_ up to roughly above 2.0, whereas OD_600_ calculated using the weighted averages was measured up to roughly 3.5, the OD_600_ of the real stationary phase. This implies that if we measure OD_600_ using only a 10 mm cuvette, we would observe a false stationary phase.

Similarly, growth curves of *P. pastoris* were also measured by two methods mentioned above and compared. The graph plotted shows a distinct difference between the OD_600_ measured using 10 mm cuvettes and the weighted averages (Figure 3B). The deviation between the graphs measured by two different methods is much more significant than those measured for *E. coli*. Graphs measured using the 10 mm cuvette exhibit an even earlier and lower stationary phase ending at an OD_600_ of just above 2.0. However, graphs measured using the weighted averages suggest that the curve is still in the exponential phase and the much later stationary phase at an OD_600_ of around 10.0. The deviation is great enough to underline the necessity of using this new method of measurement when measuring suspensions that contain particles of a large size.

### An L‐shaped cuvette and a corresponding turbidimeter are designed to simplify the measurement

**Figure 4:**
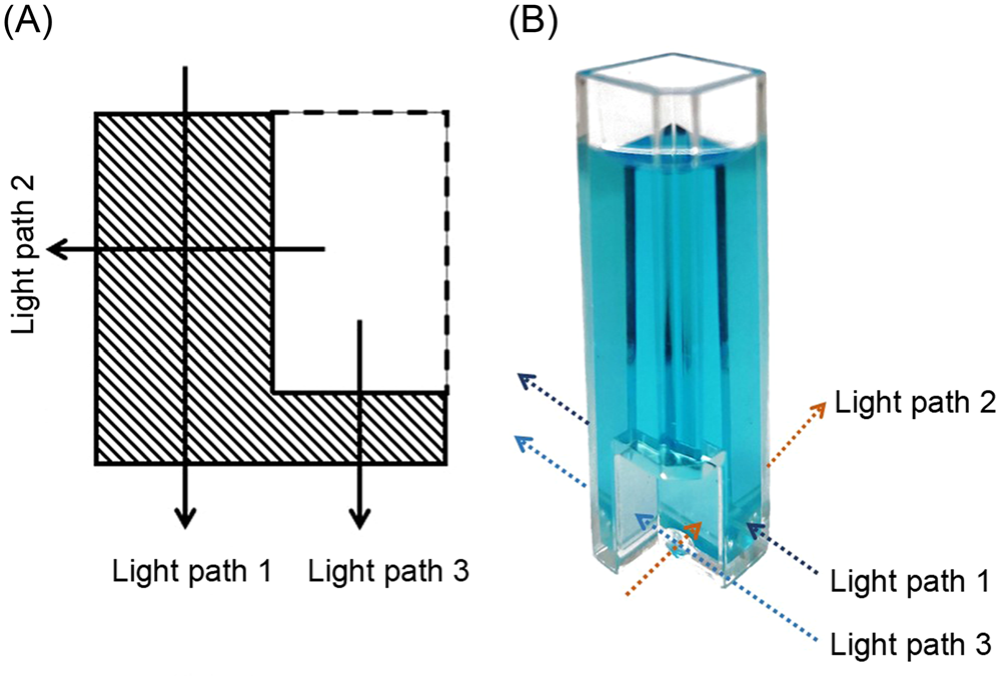
An L‐shaped cuvette is designed to simplify the measurement. The cross‐section (A) and picture (B) of an L‐shaped cuvette with three different light paths are shown.

**Figure 5:**
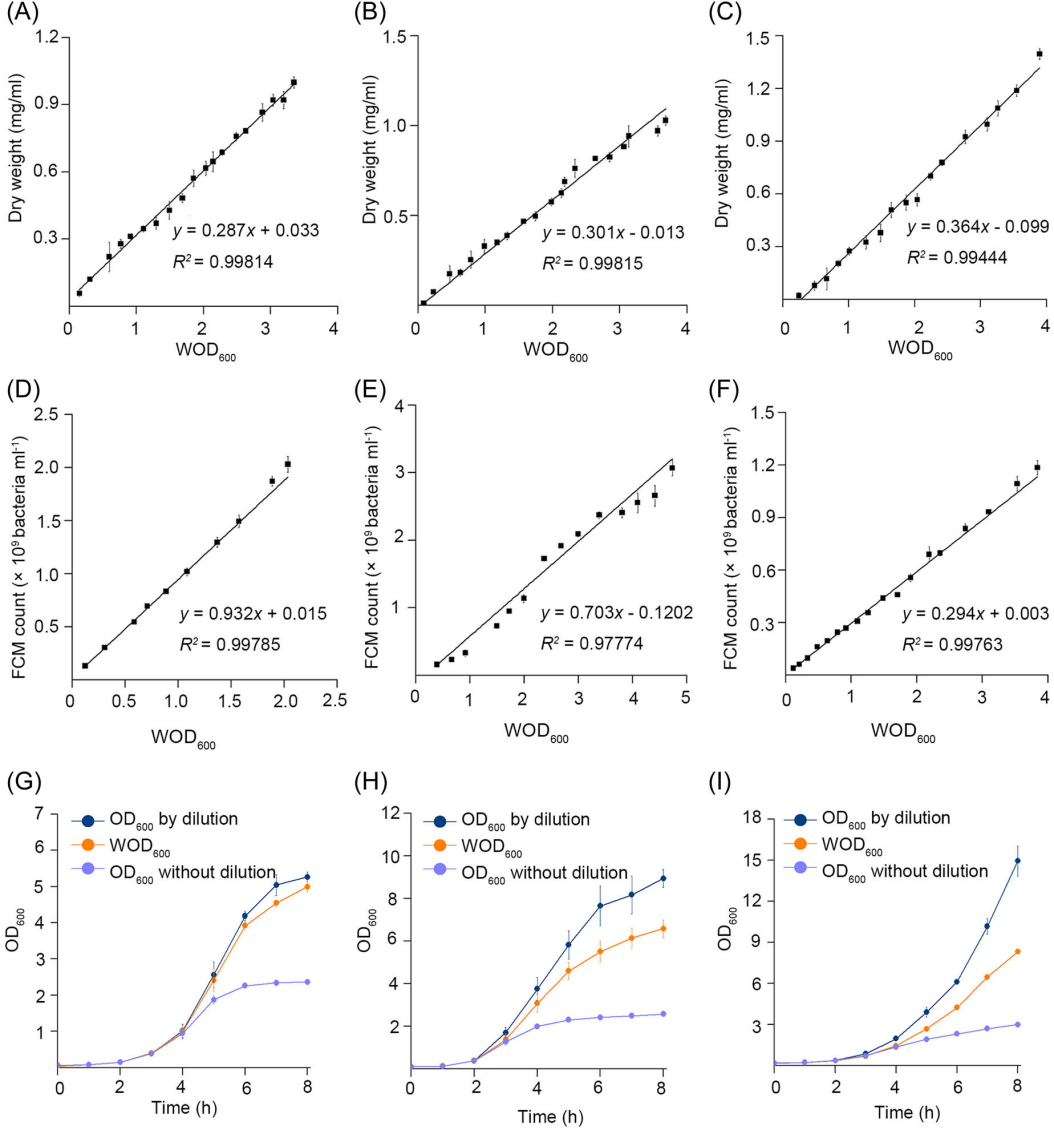
The validity of the turbidimeter is verified in *E. coli*, *S. aureus*, and *P. pastoris*. (A–C) The WOD_600_ values of *E. coli* (A), *S. aureus* (B), and *P. pastoris* (C) obtained from the turbidimeter with the L‐shaped cuvette linearly correlated with their dry weight. (D–F) The WOD_600_ values of *E. coli* (D), *S. aureus* (E), and *P. pastoris* (F) obtained by the instructions above linearly correlated with the bacterial numbers obtained by the flow cytometer (FCM for short). (G–I) Growth curves of *E. coli* (G), *S. aureus* (H), and *P. pastoris* (I) measured by the traditional method of dilution, without dilution, and using an L‐shaped cuvette and turbidimeter. Each experiment was performed in triplicate; means and standard deviations were calculated.

Measurement using the multi‐light path method is still a tedious procedure just as the dilution method. To further simplify the process, we expanded our idea to the design and production of an L‐shaped cuvette (Figure 4) and a corresponding turbidimeter. The L shape makes it possible to measure three different light paths (10, 5, and 2 mm) of a single suspension using only one cuvette. The corresponding turbidimeter shoots three beams of 600 nm light nearly simultaneously, each with a 0.5 s difference. The readings of each light path are then collected, the weighting of each value is obtained using the above‐shown equations, and the weighted averages are calculated using the function ( WOD= *w*_1_ *X*_1_ + *w*_2_ *X*_2_ + *w*_3_ *X*_3_) and displayed on the screen for the record. To verify that the WOD_600_ value obtained from the turbidimeter accurately reflects the culture density, dry weight, and bacterial numbers of *E. coli*, *S. aureus*, and *P. pastoris* cultures, a series of WOD_600_ readings were measured, respectively. Plotting all these WOD_600_ values against the dry weight gave a straight line. A linear relationship was also observed between WOD_600_ values and bacterial numbers of *E. coli*, *S. aureus*, and *P. pastoris*. These data indicate that the WOD value can accurately reflect the concentration of bacteria (Figure 5A–F). It is worth noting that *S. aureus* usually takes on the form of grape clusters^10^. This characteristic may interfere with the process of number counting by flow cytometry, thus causing a certain degree of deviation from the ideal linear relationship between the real number of the bacteria and the WOD_600_ value (Figure 5E, *R* ^2^ = 0.97774).

Growth curves for *E. coli*, *S. aureus*, and *P. pastoris* were measured using our L‐shaped cuvette and turbidimeter (Figure 5G–I). As expected, the result is much better than the conventional spectrophotometer in all three cases because conventional spectrophotometer always underestimates the cell density at high values. The curves obtained by our turbidimeter are smoother than the dilution method with smaller error bars. It is noteworthy that *P. pastoris* can grow to an OD_600_ value beyond 15 in a shake flask. The 2 mm light path still has the problem of underestimating cell density at this high value for *P. pastoris*. A new L‐shaped cuvette with 10, 5, and 1 mm has been designed and is in production to deal with cases like *P. pastoris*.

## DISCUSSION

The ideal method for measurements of microbial density should include high accuracy, convenience, and high throughput. Meeting all these three criteria has thus far been proven to be difficult. The common practice of serial dilution can give an OD value of high accuracy but is tedious and limited in throughput. Microtiter plate readers have long been used to monitor the growth of cultures in the wells of a microtiter plate, permitting high throughput measurements with ease of use^11^. However, this technique loses accuracy at high cell densities and makes dilution impossible.

In this study, we found that the multi‐light path transmission method is an alternative to dilution to achieve high accuracy. The L‐shaped cuvette and a corresponding turbidimeter offer a technique of high accuracy that is easy to use. Growth curves measured by this method have high quality from low to high cell densities and may potentially reveal some characteristics of the microbes at a later stage of growth, which may not be revealed by the microtiter plate readers. Besides OD_600_ value, other wavelengths could also be included to monitor the production of a specific metabolite. Light paths shorter than 1 mm, such as 0.5 mm and even 0.1 mm, may make measuring cell density as high as OD_600_ of 50–100 possible. The advantages of the multi‐light path transmission method also mean additional applications beyond plotting bacterial growth curves. Fermenters and bioreactors equipped with this function can monitor industrial fermentation processes in real‐time. A shaker with this capability can report any time cell density in each shaking flask. In addition, microbial ecology studies and water pollution monitoring are also potential application scenarios.

**Figure 6:**
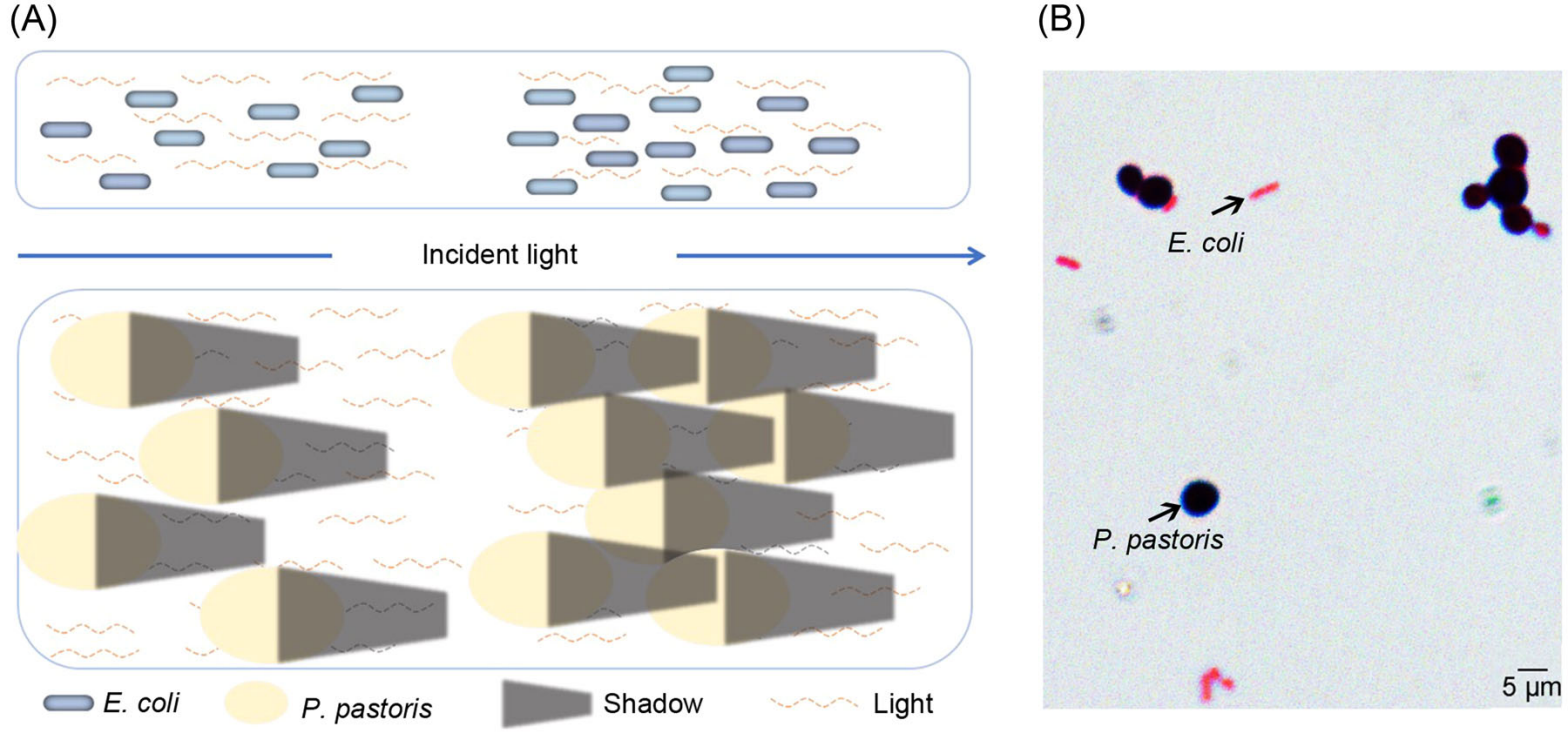
Cells of larger size have a shadow effect and thus scatter less light. (A) *E. coli* cells have a size comparable to the wavelength of 600 nm, and thus do not produce a shadowlike area on the back side of a cell. *P. pastoris* is much larger than *E. coli* and thus can cause a shadowlike area. At high densities, cells will have a greater possibility of being in the shadowlike area and would scatter less light than the cells outside the shadow, leading to a lower OD_600_ reading. The left and right parts of the image indicate cells in low and high density. (B) Size comparison of *E. coli* and *P. pastoris*.

It is worth noting that in the case of measurement of *P. pastoris*, which is larger than the cell of *E. coli*, the shorter light path readings were slightly smaller than that of the corresponding dilutions. This could be explained by the interaction between a beam of light and cells of different sizes. *E. coli* cells have a size roughly comparable to the wavelength of 600 nm. There would not be a shadow behind a cell. But in the case of *P. pastoris*, things are quite different. Its cell size is about 10–20 times larger than 600 nm, thus can cause a shadowlike area in a beam of light. The cells occasionally in the shadow would scatter less light than the cells outside the shadow, leading to a lower OD_600_ reading. In the diluted culture, however, the distance between the cells is greatly increased, and the number of cells inside shadows thus significantly decreases, giving a higher OD_600_ reading. Fortunately, this effect could be easily corrected by multiplying a coefficient based on the preliminary experiment (Figure 6A,B).

In conclusion, our study provides a novel method to measure accurate OD values of cell cultures from low to high cell density. Although our strategy lacks high throughput, based on the same principle, it is possible to design a microtiter plate with multiple light paths and the corresponding readers to achieve high accuracy, ease of use, as well as high throughput at the same time.

## MATERIALS AND METHODS

### Strains and cultures

The *E*. *coli* strain BL21 (DE3) and *S. aureus* were lab‐preserved and cultured in an incubator at 37°C with 200 rpm in sterilized Luria‐Bertani medium (LB), containing 1% peptone, 1% yeast extract, and 0.5% NaCl. The *P*. *pastoris* GS115 was a courtesy gift from Professor Hou Jin. It was inoculated in a shaker at 30°C with 260 rpm in yeast potato dextrose (YPD) medium (2% peptone, 1% yeast extract, and 2% glucose).

### Spectrophotometry in shorter light paths and dilutions

For the spectrophotometry assay, the chloramphenicol at a final concentration of 50 μg/ml was added into the overnight cultured *E*. *coli* and incubated for another 2 h to completely stop the growth of bacteria. Then, the bacteria were serially diluted into different concentrations by adding sterile water. The concentrations of the diluted bacteria were analyzed on a spectrophotometer at a wavelength of 600 nm by two different methods: (1) The bacteria were transferred into quartz cuvettes with optical path lengths of 10 mm, 5 mm, and 2 mm for measurement. Because the spectrophotometer is designed for a 10 mm light path, readings for the 5 and 2 mm are multiplied by 2 and 5, respectively, to get the real ODs. (2) The bacteria were further diluted to obtain final 1:2 and 1:5 dilutions and transferred into quartz cuvettes with an optical path length of 10 mm for measurement.

A similar procedure was also used to perform *S. aureus* and *P*. *pastoris* spectrophotometry assay with some modifications as follows. ProClin 300 at a final concentration of 3% (v/v) was added into 36‐h cultured *P*. *pastoris* and incubated for another 2 h to completely stop the growth. The yeast samples were then serially diluted into different concentrations by adding sterile water and transferred into quartz cuvettes with optical path lengths of 10 mm, 4 mm, and 1 mm for measurement. Readings for the 4 and 1 mm are multiplied by 2.5 and 10, respectively, to get the real ODs. Then the yeast aliquots were further diluted to obtain a final 1:2.5 and 1:10 dilutions and transferred into quartz cuvettes with optical path length of 1 cm for measurement.

### Growth curve detection

The growth curve was used to determine if the multi‐light path transmission method gives a more accurate reflection of the cell growth state. A single *E. coli* or *S. aureus* colony was inoculated in 15 ml LB broth and incubated overnight; 10 ml of overnight cultured *E. coli* or *S. aureus* was added into 500 ml LB medium in a conical bottle and was incubated at 37°C with shaking at 200 rpm. At time zero, 10 ml of bacteria was sampled to measure the absorbance at OD_600_ in quartz cuvettes with optical path lengths of 10 mm, 5 mm, and 2 mm, respectively. At each designated time point, this medium was measured by the same procedure. Similarly, the growth curve of yeast was also measured except the optical path lengths used were 10 mm, 4 mm, and 1 mm.

### Dry weight analysis

To analyze the dry weight of the *E*. *coli*, *S. aureus*, and yeast cells, the overnight‐cultured microorganisms were harvested. Briefly, 9 ml of each culture were transferred into centrifuge tubes and centrifuged for 15 min at 9600*g*, respectively. Then, the harvested cells were resuspended using 5 ml sterile water and then centrifuged for another 15 min at 9600*g*. Finally, the harvested cells were fully dried at 65°C overnight and the dry weight was determined using electronic balance.

### Flow cytometry analysis

Cell counting for *E. coli*, *S. aureus*, and *P*. *pastoris* was carried out on an Accuri^TM^ C6 Plus (BD Biosciences) flow cytometer; 1,000,000 events of all samples were acquired with a low flow rate of 14 μl/min. Data analysis of the cytometric files was performed by Flowjo software; a polygon gate was drawn and the events in the polygon were then counted using this software.

## AUTHOR CONTRIBUTIONS

**Hongwei Wang**: Data curation (equal); methodology (equal); writing—original draft (equal); writing—review and editing (equal). **Carina M. Gu**: Investigation (equal); methodology (equal); writing—original draft (equal). **Sujuan Xu**: Formal analysis (equal); investigation (equal); supervision (equal). **Hongfeng Wang**: Writing—original draft (supporting); writing—review and editing (supporting). **Xiaomin Zhao**: Methodology (supporting). **Lichuan Gu**: Conceptualization (lead); funding acquisition (lead); project administration (lead); writing—review and editing (equal).

## ETHICS STATEMENT

No animal or human research was involved in this study.

## CONFLICT OF INTERESTS

The authors declare no conflict of interests.

## Supporting information

Supporting information.

Supporting information.

## Data Availability

The authors confirm that the data supporting the findings of this study are available within the article and its supporting information materials.
