# Supplementary material for: Measurement of optical density of microbes by multi‐light path transmission method
Source: mLife. 2024 Dec 1;3(4):565–72. doi: 10.1002/mlf2.12147 (PMC11686084; doi:10.1002/mlf2.12147)
Supplement: Supplementary file 1 — Supporting information. [file MLF2-3-565-s002.docx]

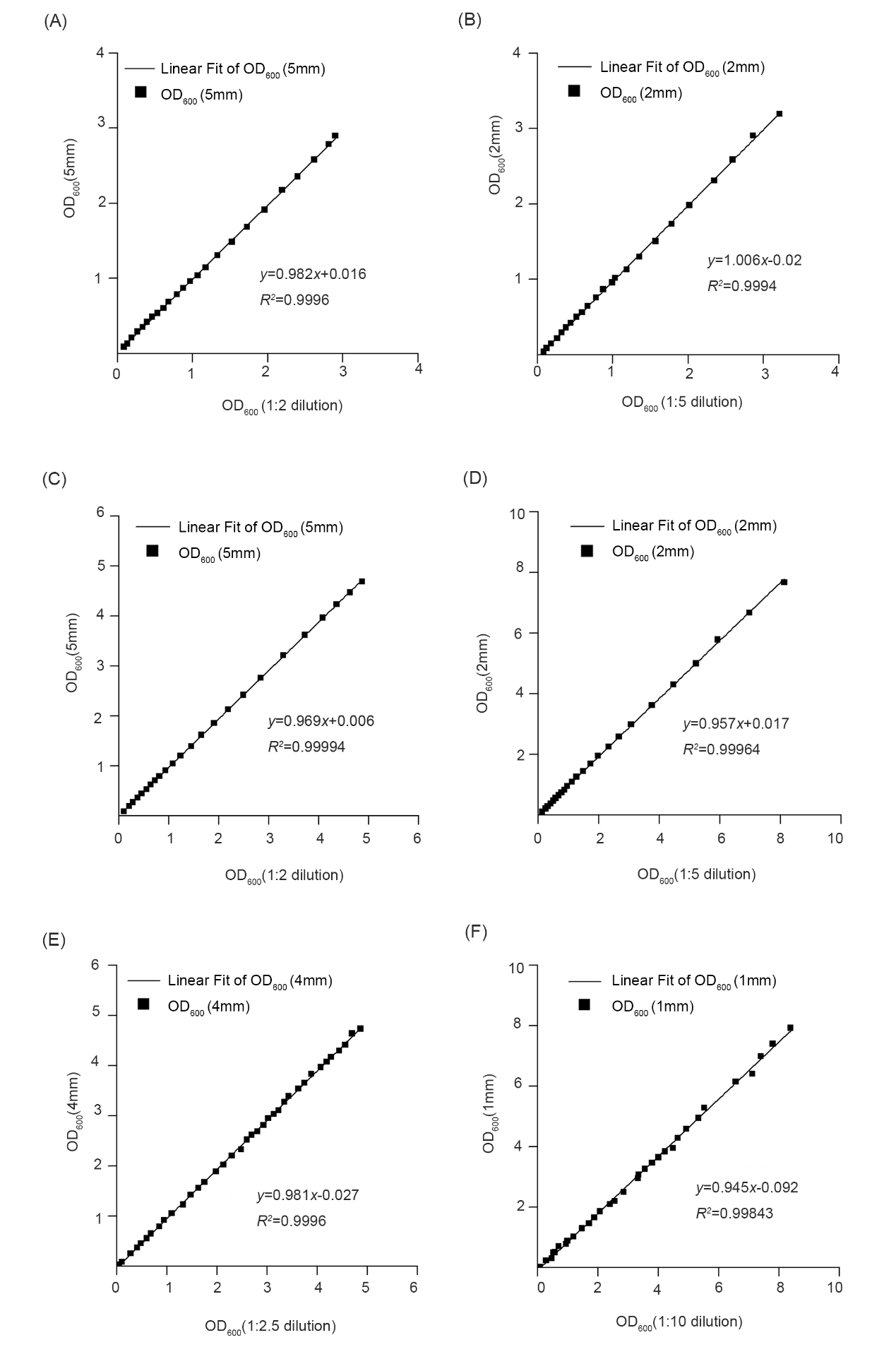


Figure S1. The shorter light path method is equivalent to the dilution method in the second repeat. (A, B) The OD_600_ values of *E. coli* culture obtained from 1:2 and 1:5 dilution is linearly correlated with 5 mm and 2 mm light paths respectively with a slope ≈ 1.0. (C, D) The OD_600_ values of *S. aureus* culture obtained from 1:2 and 1:5 dilution is also linearly correlated with 5 mm and 2 mm light paths respectively with a slope approximate to 1.0. (E, F) The OD_600_ values of *P. pastoris* obtained from 1:2.5 and 1:10 dilution is linearly correlated with 4 mm and 1 mm light paths respectively with a slope < 1.0.


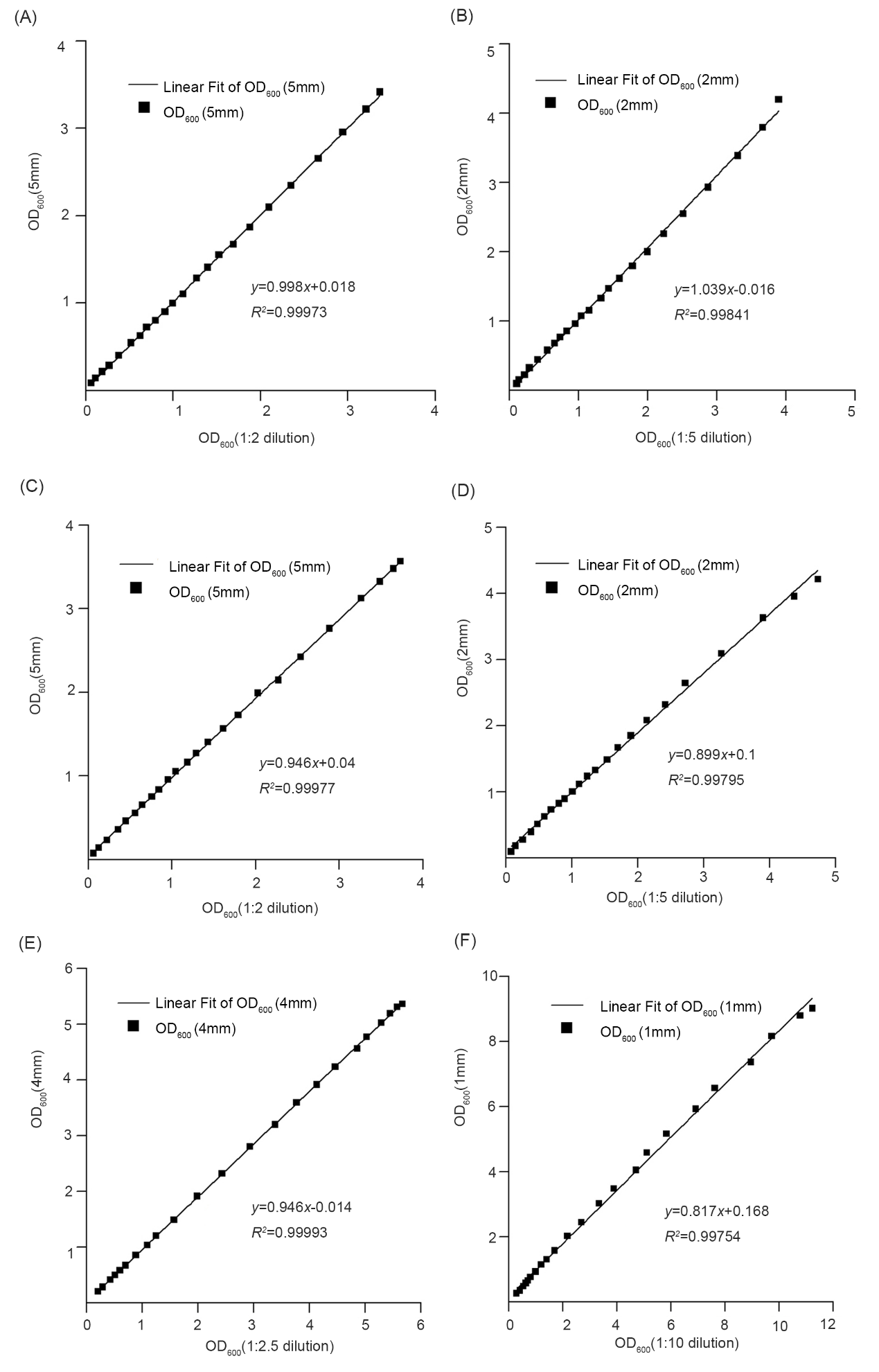


Figure S2. The shorter light path method is equivalent to the dilution method in the third repeat (A, B) The OD_600_ values of *E. coli* culture obtained from 1:2 and 1:5 dilution is linearly correlated with 5 mm and 2 mm light paths respectively with a slope ≈ 1.0. (C, D) The OD_600_ values of *S. aureus* culture obtained from 1:2 and 1:5 dilution is also linearly correlated with 5 mm and 2 mm light paths respectively with a slope approximate to 1.0. (E, F) The OD_600_ values of *P. pastoris* obtained from 1:2.5 and 1:10 dilution is linearly correlated with 4 mm and 1 mm light paths respectively with a slope < 1.0.
