# Supplementary material for: Measurement of optical density of microbes by multi‐light path transmission method
Source: mLife. 2024 Dec 1;3(4):565–72. doi: 10.1002/mlf2.12147 (PMC11686084; doi:10.1002/mlf2.12147)
Supplement: Supplementary file 2 — Supporting information. [file MLF2-3-565-s001.docx]

Table S1. OD_600_ values obtained by using shorter light paths and corresponding dilutions (*E. coli*).

| OD with conventional approach | OD with 5 mm light path | OD with 1:2 dilution | Δ OD1  (OD with 1:2 dilution - 5 mm light path) | OD with 2 mm light path | OD with 1:5 dilution | Δ OD2  (OD with 1:5 dilution - 2 mm light path) |
| --- | --- | --- | --- | --- | --- | --- |
| 2.431 | 3.980 | 3.908 | **-0.072** | 5.305 | 5.030 | **-0.275** |
| 2.354 | 3.696 | 3.658 | **-0.038** | 4.575 | 4.475 | **-0.100** |
| 2.264 | 3.396 | 3.380 | **-0.016** | 4.050 | 3.960 | **-0.090** |
| 2.190 | 3.128 | 3.124 | **-0.004** | 3.625 | 3.580 | **-0.045** |
| 2.110 | 2.880 | 2.894 | **0.014** | 3.270 | 3.245 | **-0.025** |
| 2.008 | 2.592 | 2.606 | **0.014** | 2.875 | 2.860 | **-0.015** |
| 1.905 | 2.338 | 2.352 | **0.014** | 2.550 | 2.515 | **-0.035** |
| 1.796 | 2.118 | 2.124 | **0.006** | 2.305 | 2.260 | **-0.045** |
| 1.682 | 1.914 | 1.952 | **0.038** | 2.060 | 2.045 | **-0.015** |
| 1.414 | 1.520 | 1.530 | **0.010** | 1.610 | 1.605 | **-0.005** |
| 1.326 | 1.418 | 1.440 | **0.022** | 1.500 | 1.450 | **-0.050** |
| 1.201 | 1.264 | 1.302 | **0.038** | 1.355 | 1.325 | **-0.030** |
| 1.081 | 1.126 | 1.164 | **0.038** | 1.205 | 1.185 | **-0.020** |
| 0.975 | 1.000 | 1.030 | **0.030** | 1.125 | 1.085 | **-0.040** |
| 0.853 | 0.868 | 0.898 | **0.030** | 0.985 | 0.985 | **0.000** |
| 0.743 | 0.748 | 0.770 | **0.022** | 0.855 | 0.810 | **-0.045** |
| 0.630 | 0.634 | 0.660 | **0.026** | 0.695 | 0.700 | **0.005** |
| 0.549 | 0.548 | 0.566 | **0.018** | 0.600 | 0.615 | **0.015** |
| 0.447 | 0.446 | 0.478 | **0.032** | 0.500 | 0.505 | **0.005** |
| 0.357 | 0.346 | 0.352 | **0.006** | 0.350 | 0.340 | **-0.010** |
| 0.298 | 0.292 | 0.298 | **0.006** | 0.295 | 0.290 | **-0.005** |
| 0.248 | 0.238 | 0.250 | **0.012** | 0.245 | 0.245 | **0.000** |
| 0.210 | 0.198 | 0.212 | **0.014** | 0.195 | 0.205 | **0.010** |
| 0.187 | 0.178 | 0.190 | **0.012** | 0.175 | 0.180 | **0.005** |
| 0.146 | 0.136 | 0.146 | **0.010** | 0.130 | 0.150 | **0.020** |
| 0.118 | 0.110 | 0.118 | **0.008** | 0.115 | 0.120 | **0.005** |
| 0.098 | 0.088 | 0.096 | **0.008** | 0.090 | 0.090 | **0.000** |
| 0.082 | 0.072 | 0.082 | **0.010** | 0.075 | 0.080 | **0.005** |
| 0.071 | 0.060 | 0.070 | **0.010** | 0.060 | 0.065 | **0.005** |
| 0.060 | 0.050 | 0.062 | **0.012** | 0.045 | 0.065 | **0.020** |
| 0.051 | 0.044 | 0.054 | **0.010** | 0.040 | 0.055 | **0.015** |
| 0.044 | 0.034 | 0.042 | **0.008** | 0.030 | 0.045 | **0.015** |
| 0.035 | 0.024 | 0.036 | **0.012** | 0.020 | 0.035 | **0.015** |
| 0.029 | 0.018 | 0.030 | **0.012** | 0.015 | 0.030 | **0.015** |
| 0.022 | 0.012 | 0.024 | **0.012** | 0.010 | 0.025 | **0.015** |
| 0.017 | 0.006 | 0.018 | **0.012** | 0.000 | 0.020 | **0.020** |
| 0.013 | 0.004 | 0.020 | **0.016** | 0.000 | 0.030 | **0.030** |
| 0.007 | 0.002 | 0.008 | **0.006** | 0.000 | 0.010 | **0.010** |

Table S2. OD_600_ values obtained by using shorter light paths and corresponding dilutions (*S. aureus*).

| OD with conventional approach | OD with 5 mm light path | OD with 1:2 dilution | Δ OD1  (OD with 1:2 dilution - 5 mm light path) | OD with 2 mm light path | OD with 1:5 dilution | Δ OD2  (OD with 1:5 dilution - 2 mm light path) |
| --- | --- | --- | --- | --- | --- | --- |
| 2.854 | 4.806 | 5.038 | **0.232** | 8.645 | 9.235 | **0.590** |
| 2.446 | 3.766 | 3.882 | **0.116** | 4.645 | 4.700 | **0.055** |
| 2.405 | 3.602 | 3.780 | **0.178** | 4.240 | 4.550 | **0.310** |
| 2.337 | 3.294 | 3.460 | **0.166** | 3.730 | 4.010 | **0.280** |
| 2.250 | 3.020 | 3.174 | **0.154** | 3.305 | 3.550 | **0.245** |
| 2.140 | 2.686 | 2.818 | **0.132** | 2.875 | 3.100 | **0.225** |
| 1.963 | 2.266 | 2.384 | **0.118** | 2.365 | 2.550 | **0.185** |
| 1.717 | 1.854 | 1.970 | **0.116** | 1.905 | 2.020 | **0.115** |
| 1.447 | 1.490 | 1.568 | **0.078** | 1.505 | 1.620 | **0.115** |
| 1.220 | 1.226 | 1.278 | **0.052** | 1.215 | 1.340 | **0.125** |
| 0.990 | 0.982 | 1.006 | **0.024** | 0.960 | 1.010 | **0.050** |
| 0.759 | 0.750 | 0.796 | **0.046** | 0.730 | 0.805 | **0.075** |
| 0.618 | 0.600 | 0.630 | **0.030** | 0.570 | 0.625 | **0.055** |
| 0.519 | 0.496 | 0.530 | **0.034** | 0.470 | 0.520 | **0.050** |
| 0.415 | 0.394 | 0.424 | **0.030** | 0.365 | 0.410 | **0.045** |
| 0.351 | 0.330 | 0.376 | **0.046** | 0.300 | 0.385 | **0.085** |
| 0.274 | 0.246 | 0.282 | **0.036** | 0.215 | 0.305 | **0.090** |
| 0.217 | 0.190 | 0.224 | **0.034** | 0.160 | 0.230 | **0.070** |
| 0.189 | 0.164 | 0.192 | **0.028** | 0.135 | 0.200 | **0.065** |
| 0.156 | 0.132 | 0.160 | **0.028** | 0.100 | 0.160 | **0.060** |
| 0.108 | 0.088 | 0.112 | **0.024** | 0.055 | 0.120 | **0.065** |
| 0.074 | 0.054 | 0.076 | **0.022** | 0.030 | 0.085 | **0.055** |

Table S3. OD_600_ values obtained by using shorter light paths and corresponding dilutions for (*P. pastoris*).

| OD with conventional approach | OD with 4 mm light path | OD with 1:2.5 dilution | Δ OD1 (OD with 1:2.5 dilution – 4 mm light path) | OD with 1 mm light path | OD with 1:10 dilution | Δ OD2  (OD with 1:10 dilution - 1 mm light path) |
| --- | --- | --- | --- | --- | --- | --- |
| 2.530 | 4.800 | 5.168 | **0.368** | 9.240 | 11.430 | **2.190** |
| 2.378 | 4.383 | 4.705 | **0.322** | 7.530 | 9.890 | **2.360** |
| 2.367 | 4.373 | 4.643 | **0.270** | 8.180 | 9.210 | **1.030** |
| 2.219 | 3.975 | 4.208 | **0.233** | 6.200 | 7.650 | **1.450** |
| 2.177 | 3.820 | 4.080 | **0.260** | 5.660 | 7.110 | **1.450** |
| 2.072 | 3.533 | 3.753 | **0.220** | 4.840 | 5.950 | **1.110** |
| 1.942 | 3.153 | 3.380 | **0.227** | 4.050 | 4.920 | **0.870** |
| 1.813 | 2.833 | 3.023 | **0.190** | 3.410 | 4.150 | **0.740** |
| 1.686 | 2.530 | 2.725 | **0.195** | 2.990 | 3.540 | **0.550** |
| 1.533 | 2.133 | 2.263 | **0.130** | 2.230 | 2.780 | **0.550** |
| 1.429 | 1.883 | 2.035 | **0.152** | 2.010 | 2.380 | **0.370** |
| 1.204 | 1.555 | 1.593 | **0.038** | 1.450 | 1.750 | **0.300** |
| 1.069 | 1.240 | 1.328 | **0.088** | 1.200 | 1.500 | **0.300** |
| 0.902 | 1.005 | 1.050 | **0.045** | 0.920 | 1.110 | **0.190** |
| 0.785 | 0.868 | 0.920 | **0.052** | 0.800 | 1.010 | **0.210** |
| 0.684 | 0.733 | 0.768 | **0.035** | 0.720 | 0.770 | **0.050** |
| 0.605 | 0.635 | 0.698 | **0.063** | 0.520 | 0.710 | **0.190** |
| 0.516 | 0.510 | 0.575 | **0.065** | 0.420 | 0.580 | **0.160** |
| 0.445 | 0.435 | 0.498 | **0.063** | 0.360 | 0.510 | **0.150** |
| 0.384 | 0.368 | 0.420 | **0.052** | 0.270 | 0.430 | **0.160** |
| 0.296 | 0.283 | 0.315 | **0.032** | 0.210 | 0.330 | **0.120** |
| 0.226 | 0.218 | 0.243 | **0.025** | 0.110 | 0.240 | **0.130** |
| 0.111 | 0.093 | 0.120 | **0.027** | 0.010 | 0.130 | **0.120** |
| 0.084 | 0.070 | 0.093 | **0.023** | -0.010 | 0.090 | **0.100** |
| 0.067 | 0.053 | 0.070 | **0.017** | -0.010 | 0.080 | **0.090** |
| 0.058 | 0.038 | 0.065 | **0.027** | -0.030 | 0.070 | **0.100** |
| 0.041 | 0.025 | 0.045 | **0.020** | -0.040 | 0.050 | **0.090** |
| 0.034 | 0.018 | 0.038 | **0.020** | -0.050 | 0.050 | **0.100** |
| 0.027 | 0.013 | 0.025 | **0.012** | -0.030 | 0.040 | **0.070** |
| 0.019 | 0.000 | 0.020 | **0.020** | -0.030 | 0.030 | **0.060** |
| 0.010 | -0.008 | 0.013 | **0.021** | -0.040 | 0.020 | **0.060** |
